# Triglyceride-glucose index trajectories and in-hospital mortality in heart failure: a retrospective cohort study

**DOI:** 10.3389/fcvm.2026.1945155

**Published:** 2026-09-25

**Authors:** Zongjun Hu, Xiangjun Kong, Quanwei Liu, Jianguo Chen, Xia Huang

**Affiliations:** 1 Department of Critical Care Medicine, Chongqing University Three Gorges Hospital, Chongqing, China; 2 Department of Cardiology, The Second People’s Hospital of Neijiang, Affiliated Neijiang Hospital of Southwest Medical University, Neijiang, China

**Keywords:** heart failure, in-hospital mortality, intensive care unit, trajectories, triglyceride-glucose index

## Abstract

**Background:**

High triglyceride-glucose (TyG) index levels are linked to poor prognosis in the heart failure (HF) population. However, existing studies have predominantly relied on static baseline measurements, and the association of dynamic TyG trajectories with outcomes in critically ill HF patients remains unclear. This study aimed to identify TyG index trajectories in critically ill patients with HF and evaluate their association with in-hospital mortality.

**Methods:**

This retrospective study used the MIMIC-IV (v3.1) as the derivation cohort and the eICU-CRD (v2.0) as the external validation cohort. Patients with HF admitted to the intensive care unit (ICU) were included. We applied group-based trajectory modeling (GBTM) to identify distinct TyG index trajectories during the first 7 days of ICU admission. Multivariable logistic regression was used to calculate odds ratios (ORs) and 95% confidence intervals (CIs) for the association between TyG trajectories and in-hospital mortality. Complete-case analysis, Cox regression, and E-value calculation were conducted as sensitivity analyses.

**Results:**

This analysis included 2,180 patients from MIMIC-IV and 1,433 from eICU-CRD. GBTM identified three distinct TyG trajectories: Class 1 (low-level), Class 2 (moderate-level), and Class 3 (high-level). The high-level trajectory (Class 3) exhibited the highest in-hospital mortality in both cohorts. After full adjustment, Class 3 was associated with significantly increased mortality compared with Class 1 (MIMIC-IV: OR = 1.62, 95% CI: 1.13–2.31; eICU-CRD: OR = 2.73, 95% CI: 1.59–4.67). Class 2 showed a weaker and non-significant association (MIMIC-IV: OR = 1.17, 95% CI: 0.87–1.58; eICU-CRD: OR = 1.45, 95% CI: 0.97–2.17). Sensitivity analyses showed generally consistent trends.

**Conclusion:**

In critically ill patients with HF, a high-level TyG trajectory *was* independently associated with increased in-hospital mortality, showing a level-dependent gradient across two independent cohorts.

## Introduction

Heart failure (HF) remains a major global health challenge, characterized by its rising prevalence and substantial morbidity (1, 2). Patients with HF requiring admission to the intensive care unit (ICU) represent a particularly vulnerable population, marked by clinical complexity and high mortality rates (3). While hemodynamic instability has traditionally been the primary focus in this setting, HF is increasingly reconceptualized as a complex cardiometabolic syndrome (4, 5). Central to this metabolic dysfunction is insulin resistance (IR); in the critically ill, the acute stress response exacerbates IR, precipitating a vicious cycle that accelerates systemic organ failure and worsens prognosis (6, 7).

However, accurate quantification of IR in the critical care setting remains elusive. Traditional gold standards, such as the hyperinsulinemic-euglycemic clamp, are invasive and labor-intensive. Similarly, insulin-based surrogates such as the homeostasis model assessment of insulin resistance (HOMA-IR) are frequently rendered unreliable by acute stress hyperglycemia and exogenous insulin use (8). In this context, the triglyceride–glucose (TyG) index is increasingly recognized as a validated surrogate for insulin resistance (9). Although observational studies and meta-analyses have linked elevated TyG levels to adverse outcomes in patients with heart failure, these investigations have predominantly focused on static, baseline measurements (10–13).

Reliance on a single baseline measurement, however, is inherently limited. A static measurement may not capture the sustained metabolic burden experienced during the early ICU phase. Longitudinal trajectory modeling, which characterizes patient-level patterns over time, can identify distinct TyG trajectory patterns that static metrics may overlook. Although TyG trajectories have been associated with adverse outcomes in sepsis and atrial fibrillation (14, 15), their association with mortality in critically ill HF patients remains poorly characterized.

To ensure generalizability and overcome the limitations inherent to single-center studies, this large-scale retrospective study adopted a derivation–validation design using two independent critical care databases (MIMIC-IV for derivation and eICU-CRD for external validation). By applying group-based trajectory modeling (GBTM), the analysis sought to delineate distinct longitudinal phenotypes of the TyG index during the early ICU phase and evaluate their association with in-hospital mortality.

## Methods

### Database

This retrospective study utilized a derivation-validation design using data from two distinct critical care databases. The Medical Information Mart for Intensive Care-IV (MIMIC-IV, v3.1) was employed as the derivation cohort, while the eICU Collaborative Research Database (eICU-CRD, v2.0) served as the external validation cohort. MIMIC-IV is a comprehensive single-center registry from Beth Israel Deaconess Medical Center (Boston, MA), encompassing 94,458 ICU admissions from 2008 to 2022 (16). In contrast, the eICU-CRD is a multicenter repository containing over 200,000 admissions from 208 hospitals across the United States (2014–2015) (17). Both datasets are hosted on PhysioNet (https://physionet.org/). Access was granted to author Zongjun Hu (Credential ID: 60604021) following completion of the Collaborative Institutional Training Initiative (CITI) curriculum. Both databases are publicly available de-identified datasets; as a secondary analysis of these data, no additional ethics approval or informed consent was required. The study was reported in accordance with the Strengthening the Reporting of Observational Studies in Epidemiology (STROBE) guidelines.

### Study participants

This study included patients with HF admitted to the ICU, identified from the MIMIC-IV (*n* = 2,180) and eICU-CRD (*n* = 1,433) databases. HF diagnoses were ascertained based on International Classification of Diseases, Ninth and Tenth Revision (ICD-9/ICD-10) codes (Supplementary Table S1) (18, 19). Exclusion criteria were as follows: (1) re-admissions (only the first ICU stay was analyzed); (2) insufficient longitudinal glucose data, defined as fewer than three daily mean values within the first 7 days; and (3) absence of triglyceride measurements within the same 7-day window.

### Data retrieval and variable definition

Data retrieval was conducted via Structured Query Language (SQL) scripts using Navicat Premium. Baseline laboratory parameters and vital signs were retrieved from records within the initial 24 h of ICU admission; for variables with repeated measurements during this window, arithmetic means were calculated. The retrieved variables were classified into seven principal domains: (1) Demographics (age, sex, weight); (2) Vital signs (temperature, heart rate, respiratory rate, systolic blood pressure (SBP), diastolic blood pressure (DBP), peripheral oxygen saturation (SpO2)); (3) Comorbidities (hypertension, diabetes, chronic kidney disease, cerebrovascular disease, atrial fibrillation, chronic pulmonary disease, liver disease, coronary artery disease, malignancy); (4) Laboratory data (white blood cells (WBC), platelets, hemoglobin, creatinine, blood urea nitrogen (BUN), sodium, potassium, chloride, bicarbonate); (5) Severity scores (Acute Physiology and Chronic Health Evaluation (APACHE) II for MIMIC-IV, APACHE IV for eICU-CRD); (6) Medications (angiotensin-converting enzyme inhibitors/angiotensin receptor blockers (ACEIs/ARBs), *β*-blockers, diuretics, statins, glucocorticoids, insulin); and (7) Interventions (vasoactive agents, invasive mechanical ventilation (IMV), renal replacement therapy (RRT)).

### Longitudinal TyG calculation and outcomes

Consistent with established methodologies (14, 15), consecutive 24 h windows were established commencing at ICU admission, with mean glucose values computed for each interval. Given the intermittent nature of lipid profiling in the ICU, a temporal matching protocol was implemented whereby each triglyceride measurement was paired with the mean glucose value from the corresponding 24 h window. The TyG index was derived using the formula: ln[triglyceride (mg/dL) × glucose (mg/dL)/2] (20). For patients with an ICU length of stay ≥7 days, the analysis covered the first 7 days. For those with shorter stays, all available data were utilized. The primary endpoint was all-cause in-hospital mortality during the index admission.

### Group-based trajectory modeling

Group-based trajectory modeling (GBTM) was applied to characterize distinct longitudinal TyG patterns among patients with a minimum of three recorded values (21). Analyses were performed using the lcmm package in R (22), which implements group-based trajectory modeling and inherently accommodates unbalanced longitudinal data, such that patients with different numbers of observations contribute information. Trajectories were modeled as second-order polynomials (incorporating linear and quadratic terms), with parameters estimated via maximum likelihood. Model selection was guided by the Akaike Information Criterion (AIC), Bayesian Information Criterion (BIC), sample-size-adjusted BIC (SABIC), and the average posterior probability of assignment (AvePP) (23). Final model determination balanced statistical adequacy (requiring AvePP >0.70 and a minimum class proportion of 5%) with clinical interpretability.

### Statistical analysis

Multiple imputation was applied to manage incomplete data, following the exclusion of variables with > 20% missingness (Supplementary Table S2). To describe the study population, baseline characteristics were compared across TyG trajectory groups. For continuous variables, data were presented as mean ± SD or median (IQR), contingent on the distribution; differences among groups were evaluated using one-way ANOVA or the Kruskal–Wallis test. Categorical data were expressed as frequencies (percentages) and analyzed via the chi-square test.

To evaluate potential selection bias, baseline characteristics were compared between included and excluded patients using standardized mean differences (SMDs), with SMD >0.1 considered indicative of a meaningful between-group difference. Excluded patients comprised those with fewer than three glucose measurements or missing triglyceride measurements within the first 7 days of ICU admission.

Multivariable logistic regression was employed to evaluate the association between TyG trajectory groups and in-hospital mortality, reporting odds ratios (ORs) with 95% confidence intervals (CIs). Covariates were selected based on clinical relevance, prior literature, and the change-in-estimate criterion (>10% change in OR), resulting in the inclusion of 22 variables. Collinearity diagnostics confirmed that variance inflation factors (VIFs) for all covariates were < 5 (Supplementary Table S3). Four hierarchical models were constructed: the Crude Model (unadjusted); Model 1 (adjusted for age, sex, and weight); Model 2 (additionally adjusted for temperature, respiratory rate, WBC, BUN, APACHE scores, hypertension, cerebrovascular disease, chronic pulmonary disease, liver disease, diabetes, chronic kidney disease, coronary artery disease, and atrial fibrillation); and Model 3 (further adjusted for *β*-blockers, diuretics, statins, glucocorticoids, vasoactive agents, and IMV).

Subgroup analyses were conducted by age (<65 vs. ≥65 years), sex, and key comorbidities (hypertension, diabetes, chronic kidney disease, and atrial fibrillation) to investigate potential effect modification in the MIMIC-IV cohort, with interactions assessed via likelihood ratio tests.

To verify the robustness of the primary results, three sensitivity analyses were undertaken: (1) complete-case analysis (excluding participants with missing covariates); (2) calculation of E-values to quantify the potential impact of unmeasured confounding (24); and (3) Cox regression analysis using hospital length of stay as the time variable.

All analyses were performed using R 4.6.1. A two-sided *P* value less than 0.05 was considered statistically significant.

## Results

### Study population and trajectory modeling

The initial screening identified 24,942 critically ill patients with HF (14,984 from MIMIC-IV and 9,958 from eICU-CRD). After applying exclusion criteria, 2,180 patients from the MIMIC-IV derivation cohort and 1,433 from the eICU-CRD validation cohort were retained for final analysis (Figure 1).

**Figure 1:**
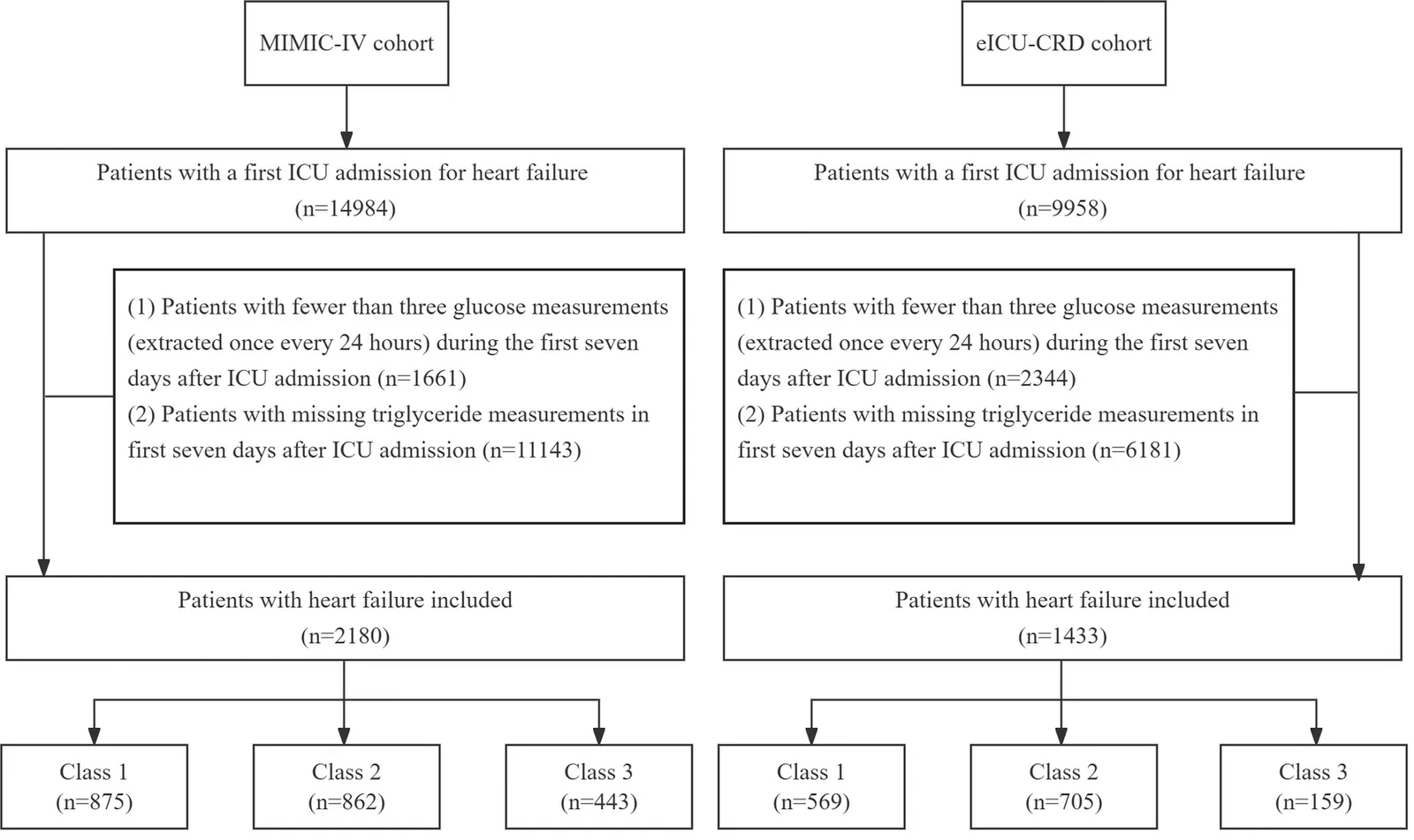
Flowchart of the selection of critically ill patients with heart failure from the MIMIC-IV and eICU-CRD databases. ICU, intensive care unit; MIMIC-IV, Medical Information Mart for Intensive Care IV; eICU-CRD, eICU Collaborative Research Database; GBTM, group-based trajectory modeling.

To characterize the excluded population, baseline characteristics of included (*n* = 2,180) and excluded (*n* = 12,804) patients were compared (Supplementary Table S4). Excluded patients were older (median 75.0 vs. 71.2 years) and exhibited a profile of greater physiological derangement, including lower temperature (36.6 vs. 36.8 °C) and diastolic blood pressure (64.0 vs. 64.4 mmHg), higher heart rate (85.0 vs. 83.8 beats/min), and slightly higher APACHE II scores (19.0 vs. 18.0), whereas other baseline characteristics were largely comparable (SMD < 0.1). In-hospital mortality was slightly higher in the excluded group (21.0% vs. 17.8%; SMD = 0.081).

Using GBTM, we identified three distinct longitudinal TyG phenotypes. The three-trajectory model was selected as the optimal solution based on model fit indices (lower AIC, BIC, and SABIC), assignment quality (AvePP >0.70), a minimum class proportion exceeding 5%, and clinical interpretability (Table 1). The identified trajectories were characterized as follows: Class 1 (low-level), comprising 40.1% of the MIMIC-IV cohort and 39.7% of the eICU-CRD cohort; Class 2 (moderate-level), comprising 39.5% (MIMIC-IV) and 49.2% (eICU-CRD); Class 3 (high-level), comprising 20.3% (MIMIC-IV) and 11.1% (eICU-CRD) (Figure 2).

**Table 1:** Model fit statistics for selecting the optimal number of latent classes in the MIMIC-IV and eICU-CRD cohorts.

Cohort	Classes	Log likelihood	AIC	BIC	SABIC	AvePP	Class proportion (%)
MIMIC-IV	2	−4,238.72	8,507.45	8,592.76	8,545.10	0.86/0.85	78.67/21.33
	3	−4,165.92	8,371.84	8,485.58	8,422.03	0.78/0.72/0.85	40.14/39.54/20.32
	4	−4,127.19	8,304.38	8,446.56	8,367.13	0.76/0.77/0.65/0.78	36.15/2.02/36.19/25.64
	5	−4,108.49	8,276.98	8,447.59	8,352.28	0.78/0.68/0.79/0.76/0.70	13.85/27.61/40.73/7.34/10.46
	6	−4,101.24	8,272.47	8,471.52	8,360.32	0.77/0.73/0.58/0.71/0.77/0.71	12.98/34.59/4.31/28.94/6.61/12.57
eICU−CRD	2	−1,149.50	2,329.00	2,408.01	2,360.36	0.92/0.81	91.97/8.03
	3	−1,111.22	2,262.44	2,367.79	2,304.26	0.76/0.76/0.79	39.71/49.20/11.10
	4	−1,077.26	2,204.52	2,336.21	2,256.79	0.75/0.65/0.90/0.78	38.94/35.80/0.91/24.35
	5	−1,067.73	2,195.47	2,353.49	2,258.19	0.75/0.53/0.63/0.86/0.78	25.75/21.00/25.05/0.98/27.22
	6	−1,055.90	2,181.79	2,366.15	2,254.97	0.74/0.53/0.77/0.60/0.88/0.76	25.61/20.94/29.87/22.12/0.70/0.77

For the selected 3-class model, AvePP and class proportions are presented in the unified order: Class 1 (low-level), Class 2 (moderate-level), Class 3 (high-level). In MIMIC-IV, model class 2 (high) and class 3 (moderate) were relabeled accordingly.

AIC, Akaike information criterion; BIC, Bayesian information criterion; SABIC, sample-size adjusted BIC; AvePP, average posterior probability; MIMIC-IV, Medical Information Mart for Intensive Care IV; eICU-CRD, eICU Collaborative Research Database.

**Figure 2:**
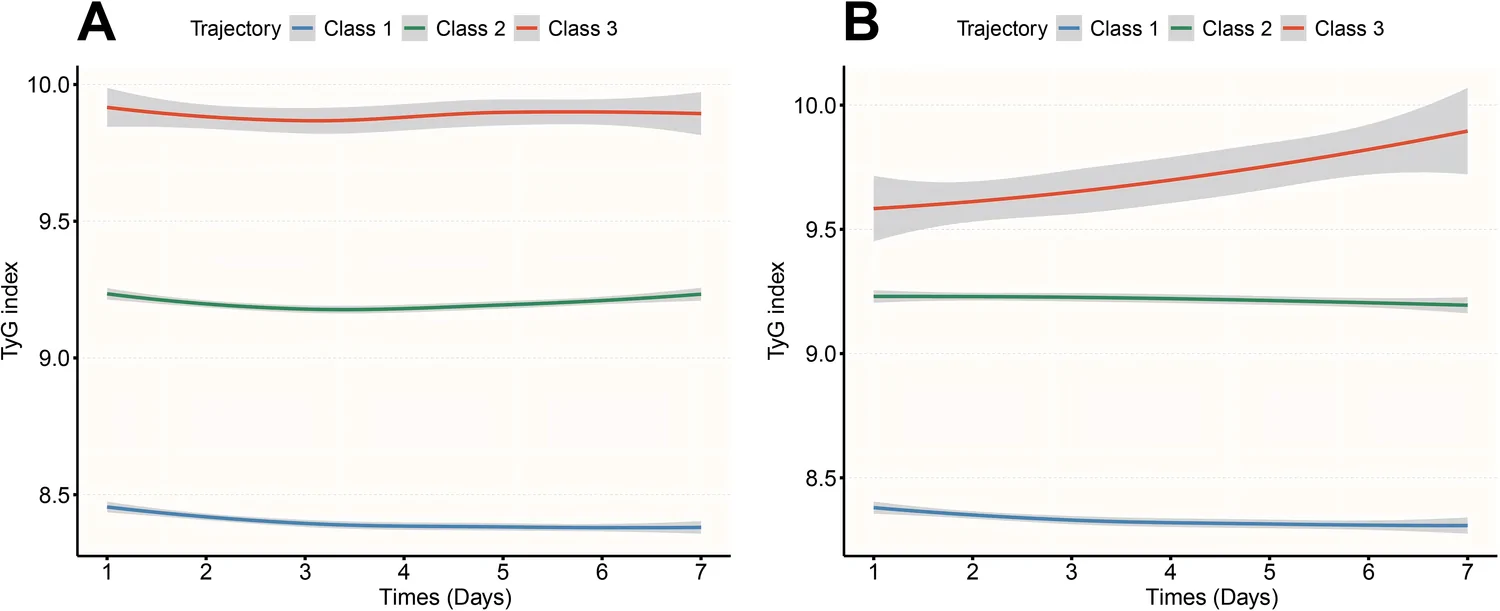
Longitudinal trajectories of the TyG index in critically ill patients with heart failure identified by group-based trajectory modeling (GBTM). **(A)** MIMIC-IV cohort (derivation cohort). **(B)** eICU-CRD cohort (validation cohort). Solid colored lines represent the estimated mean trajectories with 95% confidence intervals (shaded areas). Class 1 (blue, low-level), Class 2 (green, moderate-level), Class 3 (red, high-level). TyG, triglyceride-glucose; GBTM, group-based trajectory modeling.

### Baseline characteristics

In the MIMIC-IV cohort, compared with Class 1, Class 3 patients were younger (66.7 vs. 73.8 years) (Table 2), had higher body weight (87.7 vs. 76.7 kg), and presented with markedly greater prevalence of diabetes (64.1% vs. 21.3%) and chronic kidney disease (37.7% vs. 28.3%). Disease severity scores were highest in Class 3, with APACHE II substantially elevated (median 22.0 vs. 17.0). Therapeutic intensity was also greatest in this group: insulin use (73.4% vs. 42.9%), vasoactive agents (51.9% vs. 33.4%), invasive mechanical ventilation (64.1% vs. 31.7%), and renal replacement therapy (12.2% vs. 4.6%) were all markedly higher. Biochemically, Class 3 patients showed elevated white blood cell count (12.2 vs. 10.1 × 10^9^/L), creatinine (1.5 vs. 1.1 mg/dL), and blood urea nitrogen (28.5 vs. 22.0 mg/dL). Unadjusted in-hospital mortality was highest in Class 3 across both cohorts (27.5% in MIMIC-IV and 25.2% in eICU-CRD, both *P* < 0.001) (Table 2; Supplementary Table S5).

**Table 2:** Baseline characteristics of patients stratified by TyG index trajectory in the MIMIC-IV cohort.

Variable	Total (*n* = 2,180)	Class 1 (*n* = 875)	Class 2 (*n* = 862)	Class 3 (*n* = 443)	*P* value
Age, years	71.2 (61.0, 80.9)	73.8 (63.0, 83.4)	71.0 (61.1, 80.3)	66.7 (57.9, 76.0)	<0.001
Weight, kg	81.0 (67.9, 99.0)	76.7 (64.8, 93.1)	83.0 (69.0, 101.1)	87.7 (73.0, 105.2)	<0.001
Temperature, °C	36.8 (36.6, 37.1)	36.8 (36.6, 37.0)	36.8 (36.6, 37.2)	37.0 (36.7, 37.4)	<0.001
Heart rate, beats/min	83.8 (73.1, 97.1)	82.7 (71.7, 96.6)	83.9 (74.5, 97.1)	85.3 (74.4, 99.6)	0.024
Respiratory rate, breaths/min	20.0 (17.7, 22.9)	19.6 (17.4, 22.2)	20.1 (17.8, 22.9)	20.8 (18.2, 24.3)	<0.001
SBP, mmHg	115.7 (105.2, 130.1)	115.1 (104.8, 130.2)	116.3 (105.5, 130.7)	115.7 (106.0, 127.9)	0.637
DBP, mmHg	64.4 (57.3, 73.4)	65.1 (57.6, 75.0)	64.6 (57.4, 73.4)	63.2 (56.6, 70.5)	0.003
SpO2, %	96.6 (95.2, 97.9)	96.6 (95.2, 97.9)	96.6 (95.2, 98.0)	96.6 (95.3, 97.9)	0.726
Male, *n* (%)	1,285 (58.9)	514 (58.7)	502 (58.2)	269 (60.7)	0.680
Hypertension, *n* (%)	1,677 (76.9)	656 (75.0)	679 (78.8)	342 (77.2)	0.169
Diabetes, *n* (%)	875 (40.1)	186 (21.3)	405 (47.0)	284 (64.1)	<0.001
Chronic kidney disease, *n* (%)	725 (33.3)	248 (28.3)	310 (36.0)	167 (37.7)	<0.001
Cerebrovascular disease, *n* (%)	649 (29.8)	275 (31.4)	262 (30.4)	112 (25.3)	0.061
Atrial fibrillation, *n* (%)	1,046 (48.0)	454 (51.9)	406 (47.1)	186 (42.0)	0.002
Chronic pulmonary disease, *n* (%)	682 (31.3)	270 (30.9)	264 (30.6)	148 (33.4)	0.555
Liver disease, *n* (%)	240 (11.0)	93 (10.6)	93 (10.8)	54 (12.2)	0.670
Coronary artery disease, *n* (%)	1,184 (54.3)	459 (52.5)	466 (54.1)	259 (58.5)	0.116
Malignancy, *n* (%)	202 (9.3)	72 (8.2)	93 (10.8)	37 (8.4)	0.140
WBC, ×10⁹/L	10.9 (8.0, 15.2)	10.1 (7.7, 14.0)	11.3 (8.2, 15.6)	12.2 (8.8, 17.1)	<0.001
Platelets, ×10⁹/L	212.0 (159.0, 277.0)	209.0 (160.0, 273.8)	218.0 (163.2, 277.0)	211.0 (151.5, 284.0)	0.444
Hemoglobin, g/dL	11.5 (9.7, 13.3)	11.7 (10.0, 13.5)	11.5 (9.6, 13.3)	11.1 (9.1, 13.0)	< 0.001
Creatinine, mg/dL	1.2 (0.9, 1.8)	1.1 (0.8, 1.6)	1.2 (0.9, 1.9)	1.5 (1.0, 2.4)	<0.001
BUN, mg/dL	25.0 (17.0, 40.0)	22.0 (16.0, 35.0)	25.0 (17.0, 40.0)	28.5 (19.0, 51.0)	<0.001
Sodium, mmol/L	138.0 (135.0, 141.0)	138.0 (135.0, 141.0)	138.0 (135.0, 141.0)	137.0 (134.0, 140.0)	0.011
Potassium, mmol/L	4.3 (3.8, 4.8)	4.2 (3.8, 4.8)	4.2 (3.8, 4.8)	4.4 (3.9, 5.0)	0.004
Chloride, mmol/L	102.0 (98.0, 105.0)	102.0 (98.0, 105.0)	102.0 (98.0, 106.0)	101.0 (96.0, 104.0)	0.006
Bicarbonate, mmol/L	22.0 (19.0, 26.0)	23.0 (20.0, 26.0)	22.0 (19.0, 25.0)	22.0 (18.0, 26.0)	0.014
APACHE II	18.0 (13.0, 24.0)	17.0 (13.0, 22.0)	18.0 (13.0, 23.8)	22.0 (16.0, 28.0)	<0.001
ACEIs/ARBs, *n* (%)	235 (10.8)	115 (13.1)	93 (10.8)	27 (6.1)	<0.001
*β*-blockers, *n* (%)	902 (41.4)	399 (45.6)	367 (42.6)	136 (30.7)	<0.001
Diuretics, *n* (%)	1,038 (47.6)	416 (47.5)	418 (48.5)	204 (46.0)	0.704
Statins, *n* (%)	850 (39.0)	347 (39.7)	328 (38.1)	175 (39.5)	0.766
Glucocorticoids, *n* (%)	285 (13.1)	87 (9.9)	101 (11.7)	97 (21.9)	<0.001
Insulin, *n* (%)	1,215 (55.7)	375 (42.9)	515 (59.7)	325 (73.4)	<0.001
Vasoactive agents, *n* (%)	870 (39.9)	292 (33.4)	348 (40.4)	230 (51.9)	<0.001
RRT, *n* (%)	150 (6.9)	40 (4.6)	56 (6.5)	54 (12.2)	<0.001
IMV, *n* (%)	959 (44.0)	277 (31.7)	398 (46.2)	284 (64.1)	<0.001
In-hospital mortality, *n* (%)	388 (17.8)	116 (13.3)	150 (17.4)	122 (27.5)	<0.001

Continuous variables are presented as median (interquartile range); categorical variables as *n* (%).

Class 1 = low-level; Class 2 = moderate-level; Class 3 = high-level TyG trajectory.

SBP, systolic blood pressure; DBP, diastolic blood pressure; SpO2, peripheral oxygen saturation; WBC, white blood cells; BUN, blood urea nitrogen; APACHE II, Acute Physiology and Chronic Health Evaluation II; ACEIs/ARBs, angiotensin-converting enzyme inhibitors/angiotensin receptor blockers; RRT, renal replacement therapy; IMV, invasive mechanical ventilation.

### Association between TyG trajectories and in-hospital mortality

In the MIMIC-IV derivation cohort (Table 3), with Class 1 (low-level) as the reference, Class 3 exhibited a significantly elevated risk of in-hospital mortality in the crude model (OR = 2.49, 95% CI: 1.87–3.31, *P* < 0.001), and this association remained significant after full adjustment (OR = 1.62, 95% CI: 1.13–2.31, *P* = 0.009). Class 2 (moderate-level) showed a weaker and non-significant association after full adjustment (OR = 1.17, 95% CI: 0.87–1.58, *P* = 0.302). In the eICU-CRD validation cohort, Class 3 remained significantly associated with mortality after full adjustment (OR = 2.73, 95% CI: 1.59–4.67, *P* < 0.001), whereas Class 2 did not reach significance (OR = 1.45, 95% CI: 0.97–2.17, *P* = 0.071).

**Table 3:** Multivariable logistic regression analysis of the association between TyG trajectories and in-hospital mortality.

Cohort	Crude		Model 1		Model 2		Model 3	
	OR (95% CI)	*P* value	OR (95% CI)	*P* value	OR (95% CI)	*P* value	OR (95% CI)	*P* value
MIMIC-IV (*n* = 2,180)								
Class 1 (low)	1.00 (Reference)		1.00 (Reference)		1.00 (Reference)		1.00 (Reference)	
Class 2 (moderate)	1.38 (1.06–1.79)	0.017	1.43 (1.09–1.86)	0.009	1.31 (0.98–1.75)	0.073	1.17 (0.87–1.58)	0.302
Class 3 (high)	2.49 (1.87–3.31)	<0.001	2.75 (2.05–3.69)	<0.001	1.98 (1.40–2.80)	< 0.001	1.62 (1.13–2.31)	0.009
eICU-CRD (*n* = 1,433)								
Class 1 (low)	1.00 (Reference)		1.00 (Reference)		1.00 (Reference)		1.00 (Reference)	
Class 2 (moderate)	1.52 (1.06–2.16)	0.022	1.63 (1.13–2.34)	0.008	1.48 (0.99–2.19)	0.053	1.45 (0.97–2.17)	0.071
Class 3 (high)	3.27 (2.07–5.16)	<0.001	3.41 (2.14–5.43)	<0.001	2.97 (1.76–5.02)	< 0.001	2.73 (1.59–4.67)	< 0.001

Crude model: unadjusted. Model 1: adjusted for age, sex, and weight. Model 2: Model 1 + temperature, respiratory rate, WBC, BUN, APACHE scores (II for MIMIC-IV; IV for eICU-CRD), hypertension, cerebrovascular disease, chronic pulmonary disease, liver disease, diabetes, chronic kidney disease, coronary artery disease, and atrial fibrillation. Model 3: Model 2 + β-blockers, diuretics, statins, glucocorticoids, vasoactive agents, and IMV.

Class 1 (low-level) served as the reference group.

TyG, triglyceride-glucose index; OR, odds ratio; CI, confidence interval; WBC, white blood cells; BUN, blood urea nitrogen; APACHE, Acute Physiology and Chronic Health Evaluation; IMV, invasive mechanical ventilation.

#### Subgroup analysis

In the MIMIC-IV cohort (Figure 3), the association between Class 3 and in-hospital mortality remained stable across most subgroups, with no significant interaction observed for age, sex, hypertension, chronic kidney disease, or atrial fibrillation (all *P* for interaction > 0.05).

**Figure 3:**
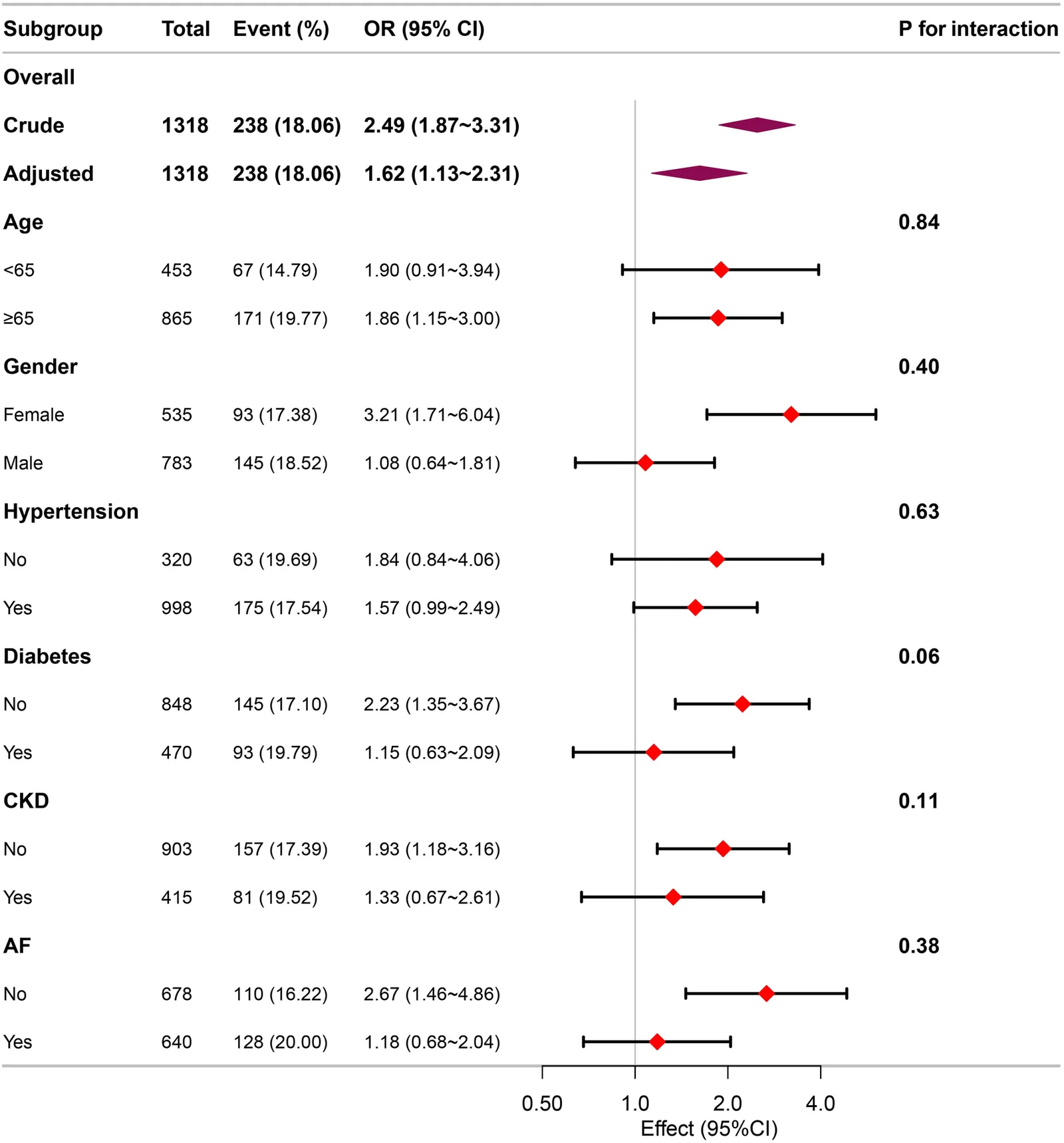
Subgroup analysis of the association between TyG trajectories and in-hospital mortality in critically ill patients with heart failure (MIMIC-IV derivation cohort). Odds ratios (ORs) and 95% confidence intervals (CIs) compare Class 3 (high-level) with Class 1 (low-level) as the reference, adjusted for age, sex, weight, temperature, respiratory rate, white blood cell count, blood urea nitrogen, APACHE II, hypertension, cerebrovascular disease, chronic pulmonary disease, liver disease, diabetes, chronic kidney disease, coronary artery disease, atrial fibrillation, *β*-blockers, diuretics, statins, glucocorticoids, vasoactive agents, and invasive mechanical ventilation, with the stratified variable excluded from the corresponding model. *P* for interaction was derived from likelihood ratio tests comparing models with and without the interaction term. TyG, triglyceride-glucose index; OR, odds ratio; CI, confidence interval; APACHE, Acute Physiology and Chronic Health Evaluation; CKD, chronic kidney disease; AF, atrial fibrillation; DM, diabetes mellitus; HTN, hypertension.

### Sensitivity analyses

In the complete-case analysis excluding participants with missing covariates (Supplementary Table S6), Class 3 remained associated with increased mortality in both cohorts (MIMIC-IV: OR = 1.68, 95% CI: 1.14–2.48, *P* = 0.008; eICU-CRD: OR = 3.02, 95% CI: 1.51–6.01, *P* = 0.001). Cox regression analysis using hospital length of stay as the time variable yielded directionally consistent results (Supplementary Table S7): with Class 1 as the reference, eICU-CRD Class 3 hazard ratio (HR) = 2.09 (95% CI: 1.30–3.36, *P* = 0.003), with MIMIC-IV showing a similar direction albeit not reaching statistical significance (HR = 1.23, 95% CI: 0.92–1.65, *P* = 0.166). E-values were 2.62 (MIMIC-IV) and 4.90 (eICU-CRD) (Supplementary Table S8).

## Discussion

In this retrospective study using a dual database derivation-validation design, we characterized the longitudinal trajectories of the TyG index among critically ill patients with heart failure. Our analysis identified three distinct TyG trajectory patterns: a low-level (Class 1), a moderate-level (Class 2), and a high-level (Class 3) trajectory. The key finding is that the high-level trajectory (Class 3) was independently associated with the highest risk of in-hospital mortality in both cohorts, whereas the moderate-level trajectory (Class 2) showed a weaker and non-significant association. These results suggest a level-dependent gradient between TyG trajectory and mortality risk. Subgroup analyses further showed that this association remained stable across most predefined subgroups.

In critically ill patients, the TyG index has been consistently associated with adverse outcomes. Liao et al. reported that the TyG index was linked to all-cause mortality in a cohort of 3,026 critically ill patients (25), and Zheng et al. demonstrated its association with in-hospital mortality in sepsis using the MIMIC-IV database (26); Wang et al. further confirmed its prognostic value in critically ill patients with coronary artery disease (27). The prognostic relevance of the TyG index also extends to heart failure. Zhou et al. showed that in patients with heart failure with preserved ejection fraction, a high TyG index was associated with increased mortality and rehospitalization (12), and Xiao et al. demonstrated an association between the TyG index at ICU admission and short-term all-cause mortality in critically ill patients with heart failure (28). However, these studies predominantly relied on static, baseline measurements, which may not fully capture the dynamic metabolic burden experienced during critical illness. In the ICU setting, dynamic changes in the TyG index during hospital stay have been associated with in-hospital and 1-year all-cause mortality (29), and trajectory-based approaches have linked distinct TyG trajectories to mortality in sepsis (14)and atrial fibrillation (15). Our study extends this line of evidence by applying GBTM-based trajectory classification specifically to critically ill patients with heart failure, and by confirming the findings in an independent validation cohort (eICU-CRD), thereby addressing the generalizability concern of single-database studies.

Patients assigned to the high-level trajectory had the highest prevalence of diabetes (64.1% vs. 21.3% in the low-level group) and the highest APACHE II scores (22.0 vs. 17.0) at baseline, suggesting a more pronounced state of insulin resistance and metabolic burden in this group. Insulin resistance impairs myocardial glucose utilization and shifts substrate preference toward fatty acid oxidation, leading to intramyocardial lipid accumulation and lipotoxicity, which promotes myocardial dysfunction and progression of heart failure (30, 31). In the ICU setting, the acute stress response—characterized by elevated catecholamines, cortisol, and inflammatory cytokines—induces stress hyperglycemia and further aggravates insulin resistance (32, 33), and a persistently high TyG trajectory may capture this sustained state of stress-related metabolic dysregulation. Accordingly, these metabolic perturbations may contribute to the higher mortality observed in the high-level group.

Subgroup analyses showed that the association between the high-level trajectory and in-hospital mortality remained stable across most predefined strata (all *P* for interaction > 0.05). Notably, the association appeared stronger in non-diabetic patients than in diabetic patients, although the interaction with diabetes did not reach statistical significance (*P* for interaction = 0.06). This differential pattern may reflect the distinct insulin resistance states between diabetic and non-diabetic patients: diabetic patients exhibit chronic insulin resistance with higher baseline TyG levels (34), which may attenuate the additional prognostic information conveyed by dynamic metabolic changes during critical illness. These findings warrant cautious interpretation and confirmation in larger cohorts.

The TyG index can be readily calculated from routinely measured glucose and triglyceride levels at no additional cost, without requiring additional blood draws or specialized assays. Trajectory analysis may therefore help identify patients with a persistently high-level TyG trajectory during the early ICU phase, who face a substantially elevated risk of in-hospital mortality, and may facilitate the characterization of dynamic metabolic status in critically ill patients with heart failure.

Several limitations warrant consideration. First, as a retrospective observational study, causal inference cannot be established, and the observed associations between TyG trajectories and in-hospital mortality may be influenced by unmeasured confounders and reverse causality. Moreover, trajectories were constructed from TyG measurements during the first 7 days of ICU admission (at least three measurements), and this exposure window partially overlaps with the outcome assessment period. Second, NT-proBNP and lactate were excluded due to high missingness and could act as unmeasured confounders; however, E-values (2.62 and 4.90) suggest that residual confounding of sufficient magnitude is unlikely. Third, excluding patients with fewer than three measurements may introduce survivor bias, as excluded patients were older with greater physiological derangement (Supplementary Table S4); nevertheless, in-hospital mortality was comparable between included and excluded patients (SMD = 0.081 < 0.1), indicating that the selection process had limited impact on the primary outcome. Given that the inclusion criteria required at least three measurements, our findings apply to patients who survive the initial acute period. In addition, ICU length of stay is correlated with disease severity and may influence trajectory estimation. Fourth, triglyceride concentrations are influenced by ICU factors (insulin, nutrition, catecholamines), and TyG dynamics are predominantly driven by glucose fluctuations (14); accordingly, our analysis focused on sustained TyG levels rather than day-to-day variability. Fifth, glucose was not measured under standardized fasting conditions, which is consistent with the real-world ICU setting, and prior studies have shown that the TyG index remains predictive under non-fasting conditions (35–37). Finally, both cohorts were derived from US critical care settings, which may limit the generalizability of our findings to other populations; future prospective multicenter studies are warranted to validate these results and to assess whether trajectory-guided metabolic management improves clinical outcomes.

## Conclusion

In critically ill patients with HF, a high-level TyG index trajectory during the early ICU period is independently associated with increased in-hospital mortality, showing a level-dependent gradient across two independent cohorts. These findings suggest that sustained high TyG levels represent a distinct high-risk metabolic profile. Prospective studies are warranted to determine whether trajectory-guided metabolic management can improve outcomes.

## Data Availability

Publicly available datasets were analyzed in this study. This data can be found here: The datasets generated and/or analyzed during the current study are available in the PhysioNet repository, https://physionet.org/. Specific datasets used include MIMIC-IV (version 3.1) [https://mimic.mit.edu/] and eICU-CRD (version 2.0) [https://eicu-crd.mit.edu/]. Access to these databases requires completion of the required online training (e.g., CITI program) and signing of a data use agreement.
