# Supplementary material for: Triglyceride-glucose index trajectories and in-hospital mortality in heart failure: a retrospective cohort study
Source: Front Cardiovasc Med. 2026 Sep 25;13:1945155. doi: 10.3389/fcvm.2026.1945155 (PMC13649691; doi:10.3389/fcvm.2026.1945155)
Supplement: Supplementary file 1 [file Table1.docx]

**Supplementary Tables**

**Table S1. ICD-9 and ICD-10 codes used to identify heart failure**

| **ICD version** | **ICD code** | **Long title** |
| --- | --- | --- |
| 9 | 398.91 | Rheumatic heart failure (congestive) |
| 9 | 402.01 | Malignant hypertensive heart disease with heart failure |
| 9 | 402.11 | Benign hypertensive heart disease with heart failure |
| 9 | 402.91 | Unspecified hypertensive heart disease with heart failure |
| 9 | 404.01 | Hypertensive heart and chronic kidney disease, malignant, with heart failure and with chronic kidney disease stage I through stage IV, or unspecified chronic kidney disease |
| 9 | 404.03 | Hypertensive heart and chronic kidney disease, malignant, with heart failure and with chronic kidney disease stage V or end stage renal disease |
| 9 | 404.11 | Hypertensive heart and chronic kidney disease, benign, with heart failure and with chronic kidney disease stage I through stage IV, or unspecified chronic kidney disease |
| 9 | 404.13 | Hypertensive heart and chronic kidney disease, benign, with heart failure and with chronic kidney disease stage V or end stage renal disease |
| 9 | 404.91 | Hypertensive heart and chronic kidney disease, unspecified, with heart failure and with chronic kidney disease stage I through stage IV, or unspecified chronic kidney disease |
| 9 | 404.93 | Hypertensive heart and chronic kidney disease, unspecified, with heart failure and with chronic kidney disease stage V or end stage renal disease |
| 9 | 428.0 | Congestive heart failure, unspecified |
| 9 | 428.1 | Left heart failure |
| 9 | 428.20 | Systolic heart failure, unspecified |
| 9 | 428.21 | Acute systolic heart failure |
| 9 | 428.22 | Chronic systolic heart failure |
| 9 | 428.23 | Acute on chronic systolic heart failure |
| 9 | 428.30 | Diastolic heart failure, unspecified |
| 9 | 428.31 | Acute diastolic heart failure |
| 9 | 428.32 | Chronic diastolic heart failure |
| 9 | 428.33 | Acute on chronic diastolic heart failure |
| 9 | 428.40 | Combined systolic and diastolic heart failure, unspecified |
| 9 | 428.41 | Acute combined systolic and diastolic heart failure |
| 9 | 428.42 | Chronic combined systolic and diastolic heart failure |
| 9 | 428.43 | Acute on chronic combined systolic and diastolic heart failure |
| 9 | 428.9 | Heart failure, unspecified |
| 10 | I09.81 | Rheumatic heart failure |
| 10 | I11.0 | Hypertensive heart disease with heart failure |
| 10 | I13.0 | Hypertensive heart and chronic kidney disease with heart failure and stage 1 through stage 4 chronic kidney disease, or unspecified chronic kidney disease |
| 10 | I13.2 | Hypertensive heart and chronic kidney disease with heart failure and with stage 5 chronic kidney disease, or end stage renal disease |
| 10 | I50.20 | Unspecified systolic (congestive) heart failure |
| 10 | I50.21 | Acute systolic (congestive) heart failure |
| 10 | I50.22 | Chronic systolic (congestive) heart failure |
| 10 | I50.23 | Acute on chronic systolic (congestive) heart failure |
| 10 | I50.30 | Unspecified diastolic (congestive) heart failure |
| 10 | I50.31 | Acute diastolic (congestive) heart failure |
| 10 | I50.32 | Chronic diastolic (congestive) heart failure |
| 10 | I50.33 | Acute on chronic diastolic (congestive) heart failure |
| 10 | I50.40 | Unspecified combined systolic (congestive) and diastolic (congestive) heart failure |
| 10 | I50.41 | Acute combined systolic (congestive) and diastolic (congestive) heart failure |
| 10 | I50.42 | Chronic combined systolic (congestive) and diastolic (congestive) heart failure |
| 10 | I50.43 | Acute on chronic combined systolic (congestive) and diastolic (congestive) heart failure |
| 10 | I50.8 | Other heart failure |
| 10 | I50.81 | Right heart failure |
| 10 | I50.810 | Right heart failure, unspecified |
| 10 | I50.811 | Acute right heart failure |
| 10 | I50.812 | Chronic right heart failure |
| 10 | I50.9 | Heart failure, unspecified |

ICD-9, International Classification of Diseases, Ninth Revision; ICD-10, Tenth Revision.

**Table S2. Variance inflation factors for covariates in the multivariable model**

| **Variable** | **MIMIC-IV VIF** | **eICU-CRD VIF** |
| --- | --- | --- |
| Age | 1.55 | 1.26 |
| Sex | 1.15 | 1.08 |
| Weight | 1.33 | 1.17 |
| Temperature | 1.12 | 1.08 |
| Respiratory rate | 1.14 | 1.14 |
| WBC | 1.10 | 1.13 |
| BUN | 1.41 | 1.27 |
| APACHE score | 1.78 | 1.68 |
| Hypertension | 1.20 | 1.06 |
| Cerebrovascular disease | 1.18 | 1.03 |
| Chronic pulmonary disease | 1.06 | 1.09 |
| Liver disease | 1.09 | 1.02 |
| Diabetes | 1.16 | 1.12 |
| Chronic kidney disease | 1.36 | 1.21 |
| Coronary artery disease | 1.15 | 1.07 |
| Atrial fibrillation | 1.17 | 1.06 |
| β-blockers | 1.23 | 1.20 |
| Diuretics | 1.06 | 1.20 |
| Statins | 1.12 | 1.20 |
| Glucocorticoids | 1.08 | 1.09 |
| Vasoactive agents | 1.58 | 1.14 |
| IMV | 1.56 | 1.42 |

All VIF < 5, no multicollinearity. MIMIC-IV, Medical Information Mart for Intensive Care IV; eICU-CRD, eICU Collaborative Research Database; VIF, variance inflation factor; WBC, white blood cell count; BUN, blood urea nitrogen; APACHE, Acute Physiology and Chronic Health Evaluation (APACHE II for MIMIC-IV and APACHE IV for eICU-CRD); IMV, invasive mechanical ventilation.

**Table S3. Extent of missing data**

| **Cohort** | **Variable** | **Missing_n** | **Missing_pct** |
| --- | --- | --- | --- |
| MIMIC-IV | Weight | 103 | 4.7 |
|  | Temperature | 49 | 2.2 |
|  | Heart rate | 4 | 0.2 |
|  | Respiratory rate | 5 | 0.2 |
|  | SBP | 19 | 0.9 |
|  | DBP | 19 | 0.9 |
|  | SpO2 | 4 | 0.2 |
|  | WBC | 30 | 1.4 |
|  | Platelets | 21 | 1 |
|  | Hemoglobin | 24 | 1.1 |
|  | Creatinine | 89 | 4.1 |
|  | BUN | 38 | 1.7 |
|  | Sodium | 177 | 8.1 |
|  | Potassium | 187 | 8.6 |
|  | Chloride | 152 | 7 |
|  | Bicarbonate | 160 | 7.3 |
| eICU-CRD | Weight | 41 | 2.9 |
|  | Temperature | 66 | 4.6 |
|  | Heart rate | 162 | 11.3 |
|  | Respiratory rate | 173 | 12.1 |
|  | SBP | 184 | 12.8 |
|  | DBP | 184 | 12.8 |
|  | SpO2 | 183 | 12.8 |
|  | WBC | 61 | 4.3 |
|  | Platelets | 56 | 3.9 |
|  | Hemoglobin | 55 | 3.8 |
|  | Creatinine | 25 | 1.7 |
|  | BUN | 24 | 1.7 |
|  | Sodium | 22 | 1.5 |
|  | Potassium | 19 | 1.3 |
|  | Chloride | 22 | 1.5 |
|  | Bicarbonate | 35 | 2.4 |
|  | APACHE score | 146 | 10.2 |

Values represent missingness before imputation. Variables with very high missingness (i.e., NT-proBNP and lactate, both > 20%) were excluded from the analysis; all remaining variables with missing values (up to 12.8% for SpO2 in eICU-CRD) were handled by multiple imputation in both cohorts (MIMIC-IV: n = 2,180; eICU-CRD: n = 1,433). MIMIC-IV, Medical Information Mart for Intensive Care IV; eICU-CRD, eICU Collaborative Research Database; NT-proBNP, N-terminal pro-brain natriuretic peptide; SBP, systolic blood pressure; DBP, diastolic blood pressure; SpO2, peripheral oxygen saturation; WBC, white blood cell count; BUN, blood urea nitrogen; APACHE, Acute Physiology and Chronic Health Evaluation (APACHE II for MIMIC-IV and APACHE IV for eICU-CRD).

**Table S4. Baseline characteristics of included and excluded patients (MIMIC-IV cohort)**

| **Variable** | **Included (n = 2,180)** | **Excluded (n = 12,804)** | **SMD** |
| --- | --- | --- | --- |
| Age, years | 71.2 (61.0, 80.9) | 75.0 (66.0, 84.0) | -0.314 * |
| Weight, kg | 81.0 (67.9, 99.0) | 80.5 (67.0, 98.0) | 0.012 |
| Temperature, °C | 36.8 (36.6, 37.1) | 36.6 (36.3, 36.9) | 0.186 * |
| Heart rate, beats/min | 83.8 (73.1, 97.1) | 85.0 (74.0, 99.0) | 0.147 * |
| Respiratory rate, breaths/min | 20.0 (17.7, 22.9) | 20.0 (17.0, 24.0) | 0.005 |
| SBP, mmHg | 115.7 (105.2, 130.1) | 115.0 (104.0, 129.0) | 0.008 |
| DBP, mmHg | 64.4 (57.3, 73.4) | 64.0 (54.0, 77.0) | 0.317 * |
| SpO2, % | 96.6 (95.2, 97.9) | 96.6 (95.0, 98.0) | -0.002 |
| Male, n (%) | 1285 (58.9%) | 7042 (55.0%) | 0.079 |
| Hypertension, n (%) | 1677 (76.9%) | 9539 (74.5%) | 0.056 |
| Diabetes, n (%) | 875 (40.1%) | 5378 (42.0%) | -0.039 |
| Chronic kidney disease, n (%) | 725 (33.3%) | 4609 (36.0%) | -0.057 |
| Cerebrovascular disease, n (%) | 649 (29.8%) | 3457 (27.0%) | 0.062 |
| Atrial fibrillation, n (%) | 1046 (48.0%) | 6402 (50.0%) | -0.040 |
| Chronic pulmonary disease, n (%) | 682 (31.3%) | 4225 (33.0%) | -0.036 |
| Liver disease, n (%) | 240 (11.0%) | 1280 (10.0%) | 0.033 |
| Coronary artery disease, n (%) | 1184 (54.3%) | 6786 (53.0%) | 0.026 |
| Malignancy, n (%) | 202 (9.3%) | 1280 (10.0%) | -0.024 |
| WBC, ×10⁹/L | 10.9 (8.0, 15.2) | 10.4 (7.5, 14.5) | 0.072 |
| Platelets, ×10⁹/L | 212.0 (159.0, 277.0) | 208.0 (155.0, 271.0) | 0.048 |
| Hemoglobin, g/dL | 11.5 (9.7, 13.3) | 11.3 (9.5, 13.1) | 0.051 |
| Creatinine, mg/dL | 1.2 (0.9, 1.8) | 1.2 (0.9, 1.9) | -0.014 |
| BUN, mg/dL | 25.0 (17.0, 40.0) | 26.0 (18.0, 43.0) | -0.078 |
| Sodium, mmol/L | 138.0 (135.0, 141.0) | 138.0 (136.0, 141.0) | -0.025 |
| Potassium, mmol/L | 4.3 (3.8, 4.8) | 4.2 (3.8, 4.7) | -0.029 |
| Chloride, mmol/L | 102.0 (98.0, 105.0) | 103.0 (99.0, 107.0) | -0.091 |
| Bicarbonate, mmol/L | 22.0 (19.0, 26.0) | 22.0 (19.0, 25.0) | 0.003 |
| APACHE II | 18.0 (13.0, 24.0) | 19.0 (14.0, 25.0) | -0.123 * |
| ACEIs/ARBs, n (%) | 235 (10.8%) | 1536 (12.0%) | -0.038 |
| β-blockers, n (%) | 902 (41.4%) | 5506 (43.0%) | -0.032 |
| Diuretics, n (%) | 1038 (47.6%) | 6274 (49.0%) | -0.028 |
| Statins, n (%) | 850 (39.0%) | 5186 (40.5%) | -0.031 |
| Glucocorticoids, n (%) | 285 (13.1%) | 1536 (12.0%) | 0.033 |
| Insulin, n (%) | 1215 (55.7%) | 6786 (53.0%) | 0.054 |
| Vasoactive agents, n (%) | 870 (39.9%) | 5186 (40.5%) | -0.012 |
| RRT, n (%) | 150 (6.9%) | 896 (7.0%) | -0.004 |
| IMV, n (%) | 959 (44.0%) | 5762 (45.0%) | -0.020 |
| In-hospital mortality, n (%) | 388 (17.8%) | 2689 (21.0%) | -0.081 |

Values are median (IQR) or n (%). SMD, standardized mean difference; SMD > 0.1 in absolute value (marked with *) indicates a meaningful between-group difference. Included = patients with at least three daily mean TyG measurements within the first 7 days of ICU admission; Excluded = patients with fewer than three glucose measurements or missing triglyceride measurements within the first 7 days. ICU, intensive care unit; TyG, triglyceride-glucose; SBP, systolic blood pressure; DBP, diastolic blood pressure; SpO2, peripheral oxygen saturation; WBC, white blood cell count; BUN, blood urea nitrogen; APACHE, Acute Physiology and Chronic Health Evaluation; ACEIs/ARBs, angiotensin-converting enzyme inhibitors/angiotensin II receptor blockers; RRT, renal replacement therapy; IMV, invasive mechanical ventilation.

**Table S5. Baseline characteristics - eICU-CRD cohort**

| **Variable** | **Total (n = 1433)** | **Class 1 low (n = 569)** | **Class 2 moderate (n = 705)** | **Class 3 high (n = 159)** | **P value** |
| --- | --- | --- | --- | --- | --- |
| Age, years | 71.0 (61.0, 79.0) | 74.0 (63.0, 83.0) | 69.0 (58.0, 77.0) | 69.0 (60.0, 78.0) | < 0.001 |
| Male, n (%) | 794 (55.4) | 326 (57.3) | 379 (53.8) | 89 (56.0) | 0.446 |
| Weight, kg | 86.1 (70.4, 105.0) | 81.5 (65.9, 96.0) | 88.8 (72.5, 109.3) | 95.7 (78.1, 115.1) | < 0.001 |
| Heart rate, beats/min | 82.9 (74.0, 94.0) | 81.8 (72.5, 93.8) | 83.4 (75.3, 94.8) | 82.7 (74.7, 92.7) | 0.067 |
| Respiratory rate, breaths/min | 20.3 (17.9, 23.1) | 20.1 (18.0, 23.0) | 20.5 (18.0, 23.3) | 20.2 (17.7, 22.7) | 0.417 |
| SBP, mmHg | 119.7 (108.2, 132.9) | 118.3 (107.0, 133.0) | 120.4 (109.8, 133.0) | 119.3 (108.8, 131.8) | 0.132 |
| DBP, mmHg | 66.0 (58.7, 74.0) | 67.0 (59.5, 74.7) | 65.8 (58.7, 73.9) | 64.0 (56.2, 72.3) | 0.021 |
| SpO2, % | 97.0 (95.7, 98.2) | 97.2 (95.8, 98.2) | 97.0 (95.6, 98.2) | 96.8 (95.9, 98.2) | 0.724 |
| Hypertension, n (%) | 390 (27.2) | 146 (25.7) | 201 (28.5) | 43 (27.0) | 0.523 |
| Diabetes, n (%) | 590 (41.2) | 138 (24.3) | 347 (49.2) | 105 (66.0) | < 0.001 |
| Chronic kidney disease, n (%) | 298 (20.8) | 110 (19.3) | 141 (20.0) | 47 (29.6) | 0.015 |
| Cerebrovascular disease, n (%) | 154 (10.7) | 59 (10.4) | 77 (10.9) | 18 (11.3) | 0.922 |
| Atrial fibrillation, n (%) | 325 (22.7) | 161 (28.3) | 137 (19.4) | 27 (17.0) | < 0.001 |
| Chronic pulmonary disease, n (%) | 311 (21.7) | 117 (20.6) | 149 (21.1) | 45 (28.3) | 0.098 |
| Liver disease, n (%) | 27 (1.9) | 14 (2.5) | 10 (1.4) | 3 (1.9) | 0.397 |
| Coronary artery disease, n (%) | 426 (29.7) | 157 (27.6) | 224 (31.8) | 45 (28.3) | 0.246 |
| Malignancy, n (%) | 136 (9.5) | 65 (11.4) | 56 (7.9) | 15 (9.4) | 0.109 |
| WBC, ×10⁹/L | 10.8 (8.0, 14.4) | 10.1 (7.5, 13.1) | 11.4 (8.6, 15.0) | 11.8 (8.9, 16.1) | < 0.001 |
| Platelets, ×10^9/L | 211.0 (162.0, 267.0) | 207.0 (152.0, 265.0) | 212.0 (168.0, 267.0) | 217.0 (167.5, 281.0) | 0.198 |
| Hemoglobin, g/dL | 11.8 (10.0, 13.6) | 11.9 (10.2, 13.5) | 11.7 (9.9, 13.6) | 11.6 (10.0, 13.9) | 0.909 |
| Creatinine, mg/dL | 1.3 (0.9, 2.0) | 1.3 (0.9, 2.0) | 1.3 (1.0, 2.0) | 1.5 (1.1, 2.3) | 0.015 |
| BUN, mg/dL | 26.0 (18.0, 43.0) | 25.0 (18.0, 42.0) | 26.0 (18.0, 43.0) | 32.4 (19.5, 44.0) | 0.033 |
| Sodium, mmol/L | 138.0 (135.0, 141.0) | 138.0 (135.0, 141.0) | 138.0 (135.0, 141.0) | 138.0 (134.0, 141.0) | 0.920 |
| Potassium, mmol/L | 4.2 (3.8, 4.7) | 4.1 (3.7, 4.6) | 4.2 (3.7, 4.7) | 4.3 (3.8, 5.0) | 0.017 |
| Chloride, mmol/L | 102.0 (98.0, 106.0) | 102.0 (98.0, 106.0) | 102.0 (98.0, 106.0) | 101.0 (97.0, 105.0) | 0.017 |
| Bicarbonate, mmol/L | 25.0 (21.7, 29.0) | 25.0 (22.0, 29.0) | 25.0 (21.0, 28.0) | 25.0 (22.0, 30.0) | 0.183 |
| APACHE IV | 60.0 (47.0, 74.2) | 59.0 (46.0, 71.0) | 61.0 (47.0, 75.0) | 65.0 (52.5, 82.0) | < 0.001 |
| ACEIs/ARBs, n (%) | 111 (7.7) | 50 (8.8) | 57 (8.1) | 4 (2.5) | 0.029 |
| β-blockers, n (%) | 428 (29.9) | 180 (31.6) | 215 (30.5) | 33 (20.8) | 0.026 |
| Diuretics, n (%) | 561 (39.1) | 212 (37.3) | 286 (40.6) | 63 (39.6) | 0.481 |
| Statins, n (%) | 260 (18.1) | 106 (18.6) | 134 (19.0) | 20 (12.6) | 0.153 |
| Glucocorticoids, n (%) | 120 (8.4) | 34 (6.0) | 64 (9.1) | 22 (13.8) | 0.004 |
| Insulin, n (%) | 414 (28.9) | 124 (21.8) | 237 (33.6) | 53 (33.3) | < 0.001 |
| Vasoactive agents, n (%) | 255 (17.8) | 103 (18.1) | 120 (17.0) | 32 (20.1) | 0.633 |
| RRT, n (%) | 68 (4.7) | 22 (3.9) | 41 (5.8) | 5 (3.1) | 0.160 |
| IMV, n (%) | 417 (29.1) | 114 (20.0) | 228 (32.3) | 75 (47.2) | < 0.001 |
| In-hospital mortality, n (%) | 188 (13.1) | 53 (9.3) | 95 (13.5) | 40 (25.2) | < 0.001 |

Values are median (IQR) or n (%). P values from Kruskal-Wallis test (continuous variables) or chi-square test (categorical variables). Class 1=low, Class 2=moderate, Class 3=high. SBP, systolic blood pressure; DBP, diastolic blood pressure; SpO2, peripheral oxygen saturation; WBC, white blood cell count; BUN, blood urea nitrogen; APACHE, Acute Physiology and Chronic Health Evaluation (APACHE IV for eICU-CRD); ACEIs/ARBs, angiotensin-converting enzyme inhibitors/angiotensin II receptor blockers; RRT, renal replacement therapy; IMV, invasive mechanical ventilation.

**Table S6. Complete-case sensitivity analysis**

| **Cohort** | **N** | **Events** | **Class** | **OR** | **P** |
| --- | --- | --- | --- | --- | --- |
| MIMIC-IV (complete-case) | 1970/2180 | 341 | Class 2 vs 1 | 1.20 (0.87–1.66) | 0.260 |
| MIMIC-IV (complete-case) | 1970/2180 | 341 | Class 3 vs 1 | 1.68 (1.14–2.48) | 0.008 |
| eICU-CRD (complete-case, pre-imputation) | 1068/1433 | 144 | Class 2 vs 1 | 1.46 (0.89–2.41) | 0.140 |
| eICU-CRD (complete-case, pre-imputation) | 1068/1433 | 144 | Class 3 vs 1 | 3.02 (1.51–6.01) | < 0.001 |
| eICU-CRD (imputed) | 1433/1433 | 188 | Class 2 vs 1 | 1.45 (0.97–2.17) | 0.071 |
| eICU-CRD (imputed) | 1433/1433 | 188 | Class 3 vs 1 | 2.73 (1.59–4.67) | < 0.001 |

Complete-case analysis excluding participants with missing covariates. Adjusted for age, sex, weight, temperature, respiratory rate, WBC, BUN, APACHE scores, hypertension, cerebrovascular disease, chronic pulmonary disease, liver disease, diabetes, chronic kidney disease, coronary artery disease, atrial fibrillation, β-blockers, diuretics, statins, glucocorticoids, vasoactive agents, and IMV. Class 1 = reference. MIMIC-IV, Medical Information Mart for Intensive Care IV; eICU-CRD, eICU Collaborative Research Database; OR, odds ratio; WBC, white blood cell count; BUN, blood urea nitrogen; APACHE, Acute Physiology and Chronic Health Evaluation (APACHE II for MIMIC-IV and APACHE IV for eICU-CRD); IMV, invasive mechanical ventilation.

**Table S7. Cox regression sensitivity analysis**

| **Cohort** | **Analysis** | **Class** | **HR** | **P** |
| --- | --- | --- | --- | --- |
| MIMIC-IV | Full follow-up | Class 2 vs 1 | 0.92 (0.72–1.19) | 0.536 |
|  | Full follow-up | Class 3 vs 1 | 1.23 (0.92–1.65) | 0.166 |
| eICU-CRD | Full follow-up | Class 2 vs 1 | 1.23 (0.86–1.77) | 0.250 |
|  | Full follow-up | Class 3 vs 1 | 2.09 (1.30–3.36) | 0.003 |

Hazard ratios from Cox regression with hospital length of stay as the time variable, adjusted for age, sex, weight, temperature, respiratory rate, WBC, BUN, APACHE scores, hypertension, cerebrovascular disease, chronic pulmonary disease, liver disease, diabetes, chronic kidney disease, coronary artery disease, atrial fibrillation, β-blockers, diuretics, statins, glucocorticoids, vasoactive agents, and IMV. Class 1 = reference. MIMIC-IV, Medical Information Mart for Intensive Care IV; eICU-CRD, eICU Collaborative Research Database; HR, hazard ratio; WBC, white blood cell count; BUN, blood urea nitrogen; APACHE, Acute Physiology and Chronic Health Evaluation (APACHE II for MIMIC-IV and APACHE IV for eICU-CRD); IMV, invasive mechanical ventilation.

**Table S8. E-values for TyG trajectories and in-hospital mortality**

| **Cohort** | **Comparison** | **OR** | **E-value** | **E-value (lower confidence limit)** |
| --- | --- | --- | --- | --- |
| MIMIC-IV | Class 3 (high) vs Class 1 (low) | 1.62 (1.13–2.31) | 2.62 | 1.51 |
| eICU-CRD | Class 3 (high) vs Class 1 (low) | 2.73 (1.59–4.67) | 4.90 | 2.56 |

E-values for Class 3 (high) vs Class 1 (low), Model 3. MIMIC-IV, Medical Information Mart for Intensive Care IV; eICU-CRD, eICU Collaborative Research Database; OR, odds ratio; CI, confidence interval.
